# Coinfection of Malaria, Parvovirus, Epstein-Barr Virus, Respiratory Syncytial Virus, and Lyme Disease in a Returning Traveller: A Case Report

**DOI:** 10.7759/cureus.111539

**Published:** 2026-06-26

**Authors:** Sadia Shoaib, Mahmoud A Mohamed, Saddam H Abbasi, Momina Javed, Kashif Shaikh, Rifat Alam, Aniqua Tareen

**Affiliations:** 1 General Medicine, Luton and Dunstable Hospital, Luton, GBR; 2 Acute Medicine, Luton and Dunstable Hospital, Luton, GBR; 3 Internal Medicine, Luton and Dunstable Hospital, Luton, GBR

**Keywords:** co-infection, epstein-barr virus, haemolysis, lyme disease, malaria

## Abstract

We report the case of a 36-year-old woman presenting with fever, jaundice, and haemolysis after recent travel to Ghana. She was ultimately diagnosed with concurrent infections: *Plasmodium falciparum *(*P. falciparum*), parvovirus B19, Epstein-Barr virus (EBV), respiratory syncytial virus (RSV), and *Borrelia burgdorferi* (Lyme disease). Her course was complicated by profound haemolysis, acute kidney injury, and persistent fever despite antimalarial therapy. This case highlights the diagnostic and therapeutic challenges of polymicrobial infections in returning travellers, the overlapping mechanisms of haemolysis, and the importance of a multidisciplinary approach to care.

## Introduction

Fever in a returning traveller represents one of the more challenging scenarios in clinical medicine. Malaria must always be considered promptly due to its potential severity, particularly infections caused by *Plasmodium falciparum* (*P. falciparum*). Compared with other malaria species, *P. falciparum* can cause rapid progression to severe complications, including cerebral malaria, multi-organ failure, and death, making timely diagnosis and treatment essential. However, febrile illness in travellers is not always due to a single pathogen. Individuals may acquire multiple infections through varied exposures during travel or experience reactivation of latent infections triggered by the physiological stress of acute illness, such as dehydration, fever, or immune suppression [1].

Coinfections further complicate clinical evaluation. When multiple pathogens coexist, overlapping symptoms can obscure the contribution of individual infections, a phenomenon sometimes referred to as “diagnostic overshadowing.” Management also becomes more nuanced, as clinicians must select treatments that are both effective and safe, especially when therapies carry their own risks [2]. While coinfections are relatively common in endemic, resource-limited regions, they are less frequently recognised in high-income countries and may be underappreciated in routine clinical practice [3]. Estimates suggest that 5%-20% of travellers presenting with febrile illness may have more than one pathogen contributing to their symptoms, particularly when malaria is involved. Common combinations include malaria + dengue, malaria + bacterial sepsis (*Salmonella*, *Escherichia coli*), malaria + parvovirus B19, or malaria + Epstein-Barr virus (EBV) [4].

Malaria continues to impose a significant global health burden, with an estimated 249 million cases and 608,000 deaths worldwide in 2022 [5]. In non-endemic regions, malaria is most often seen in travellers, especially those visiting friends and relatives, who may not have taken appropriate prophylaxis [6]. Viral infections such as parvovirus B19 and EBV can remain latent in the body and may reactivate under physiological stress, including acute febrile illness, due to temporary immune dysregulation, while seasonal respiratory viruses like respiratory syncytial virus (RSV) may further complicate the clinical picture [7-9]. Even vector-borne infections like Lyme disease, common in temperate climates, can further complicate diagnosis by producing overlapping or nonspecific symptoms [10].

Given these complexities, clinicians must employ a systematic approach to workup, incorporating travel history, exposure risk, timing and pattern of symptoms, and targeted laboratory testing. Awareness of common coinfections, including malaria with viral reactivations or bacterial infections, can guide investigations, ensure timely identification of high-risk pathogens such as *P. falciparum*, and facilitate safe, effective management in patients presenting with multi-pathogen illness.

## Case presentation

A 36-year-old woman presented with five days of fever, generalised aches, nausea and headaches. She had returned five days earlier from a three-week stay with relatives in Ghana, where she had not taken malaria prophylaxis. Further history revealed significant outdoor exposure during her stay, including frequent insect bites and close contact with family members and the local community, providing several potential opportunities for infectious exposures. In the days leading up to presentation, she described worsening fatigue and noticed yellowing of her eyes (jaundice) but denied cough, rash, abdominal pain, or neck stiffness. Her past medical history was notable only for well-controlled hypertension; she took no regular medications and had no known immunodeficiency.

On examination, she appeared unwell: febrile (38.5°C), tachycardic (108 beats/minute), and borderline hypotensive (102/64 mmHg). She was visibly jaundiced with scleral icterus. The abdomen was soft, but a palpable spleen tip was noted; no hepatomegaly, rash or lymphadenopathy was present.

Initial blood tests were striking. C-reactive protein was >400 mg/L and ferritin ~12,000 μg/L (marked inflammation). Liver function tests showed bilirubin at 73 μmol/L, alanine aminotransferase (ALT) at 178 U/L, and alkaline phosphatase (ALP) at 244 U/L, and renal function was impaired (urea 24.1 mmol/L, creatinine 288 μmol/L) (Table 1). Haematology revealed severe haemolysis: lactate dehydrogenase (LDH) 2935 U/L and a falling haemoglobin (down to 69 g/L) despite a high reticulocyte count (337×10^9/L), indicating active red-cell destruction. A peripheral blood smear confirmed heavy *P. falciparum* parasitaemia (37.7% of erythrocytes).

**Table 1:** Blood test results CRP, C-reactive protein; ALT, alanine aminotransferase; ALP, alkaline phosphatase

Test	Result	Reference Value
CRP	413mg/L	0 - 4.9 mg/L
Ferritin	12,039	13 - 150 µg/L
Bilirubin	73 µmol/L	0 - 20 µmol/L
ALT	178 U/L	0 - 32 U/L
ALP	244 U/L	30 - 130 U/L
Urea	24.1 mmol/L	2.5 - 7.8 mmol/L
Creatinine	288 µmol/L	44 - 80 µmol/L

**Figure 1:**
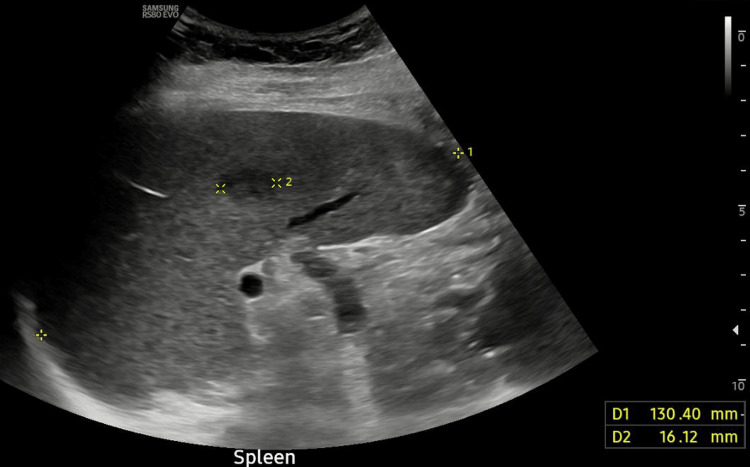
USG of the abdomen showing splenomegaly with a slightly hyperechoic area.

Imaging with abdominal ultrasound demonstrated splenomegaly and gallbladder sludge (Figure 1). A CT scan of the thorax, abdomen, and pelvis showed minimal pericholecystic fluid but no evidence of biliary obstruction. 

She was diagnosed with severe falciparum malaria and started immediately on intravenous artesunate (the recommended first-line treatment for severe malaria). After 72 hours of IV therapy, she was switched to oral artemether-lumefantrine to complete treatment. The parasites cleared on follow-up smears, but her fever persisted, and inflammatory markers remained elevated. Unexpectedly, her anaemia continued to worsen rather than improve.

Because her clinical course was not typical for treated malaria, the diagnostic workup was broadened. Blood cultures grew coagulase-negative *Staphylococcus *(likely skin contaminant), for which empirical ceftriaxone was started. Respiratory viral PCR unexpectedly detected RSV (though adult RSV often causes only mild illness). Serological tests showed both IgM and IgG antibodies to parvovirus B19 and to EBV, consistent with recent infection or reactivation of each. Lyme serology (indirect fluorescent antibody assay (immunoblot) was positive for IgM but negative for IgG. Broad screening for other infections (HIV; hepatitis A-E; cytomegalovirus (CMV); toxoplasmosis; brucellosis; *Rickettsia *infection; Q fever) was negative (Table 2).

**Table 2:** Viral and bacterial serology

Test	Results	Reference Value
Epstein–Barr virus IgG/IgM antibody	Positive	Negative
Lyme's disease serology: *Borrelia *IgM	Positive	Negative
Respiratory syncytial virus	Positive	Negative
Parvovirus 19 IgG/IgM	Positive	Negative
*Brucella s*erology	Negative	Negative
Severe acute respiratory syndrome coronavirus 2 (SARS-CoV-2)	Negative	Negative
Influenza A virus	Negative	Negative
Influenza B virus	Negative	Negative
Cytomegalovirus	Negative	Negative
*Rickettsia *serology	Negative	Negative

Over the next few days, her haemolysis worsened: haemoglobin stayed low, and LDH remained very high. Direct antiglobulin (Coombs) testing became strongly positive for C3d and negative for IgG, the pattern seen in cold agglutinin autoimmune haemolytic anaemia. Cold agglutinins were confirmed on an eluate from her red cells. She required transfusion of three units of packed red blood cells. Because immune-mediated haemolysis was suspected, prednisolone was started and then tapered, along with folic acid supplementation. Electrolyte disturbances (hypophosphataemia, hypomagnesaemia) were corrected.

The patient’s care was coordinated with infectious diseases and haematology specialists. Her antibiotics were changed to doxycycline. She was later moved to a tertiary centre because of persistent haemolysis. After a week, when her haemoglobin levels stabilised, she was discharged from the facility. She was followed up as an outpatient by haematology, and her blood counts and organ function eventually normalised (haemoglobin increased to 123 mg/dL on follow-up).

## Discussion

This case illustrates several important and interrelated learning points regarding the evaluation of fever in returning travellers, the interpretation of infectious serology, and the pathophysiology of severe haemolysis in polymicrobial infection.

Coinfections in travellers are increasingly recognised, particularly among patients returning from tropical or resource-limited settings. Multiple infectious aetiologies may coexist in febrile travellers, and malaria can occur concurrently with bacterial or viral infections, thereby complicating diagnosis and management [2,3]. The most commonly reported malaria-associated coinfections include dengue virus, *Salmonella *species, HIV, rickettsial disease, and viral infections such as EBV or parvovirus B19. In endemic settings, coinfection rates may be even higher due to repeated exposure to multiple pathogens and underlying immune activation. The diagnostic challenge lies in the fact that many of these infections share overlapping manifestations, including fever, cytopenias, hepatitis, splenomegaly, and elevated inflammatory markers. Once malaria is identified, there is a risk of “premature diagnostic closure,” where clinicians may incorrectly attribute all abnormalities to malaria alone. Persistent fever, worsening anaemia, progressive organ dysfunction, or laboratory features disproportionate to the degree of parasitaemia should prompt reassessment and investigation for additional processes. In this patient, the degree of inflammation and prolonged haemolysis appeared disproportionate even for severe falciparum malaria, justifying expanded testing that subsequently revealed evidence of multiple concomitant infections.

The patient fulfilled criteria for severe falciparum malaria given the very high parasitaemia (37.7%), acute kidney injury, jaundice, and severe haemolysis [5]. Severe malaria is associated with mortality rates approaching 10%-20% even with treatment [11]. Haemolysis in malaria is multifactorial and results from direct rupture of parasitised erythrocytes, immune-mediated destruction of non-parasitised erythrocytes, splenic sequestration, oxidative stress, membrane damage, and complement activation. Interestingly, the degree of anaemia in falciparum malaria often exceeds what would be expected purely from parasite burden because non-infected erythrocytes are also prematurely cleared. Another important contributor may have been post-artesunate delayed haemolysis (PADH), which is increasingly recognised following intravenous artesunate therapy and typically occurs one to three weeks after treatment initiation [12]. Proposed mechanisms include delayed splenic clearance of once-infected erythrocytes (“pitted” erythrocytes) and persistent immune activation. Studies suggest PADH occurs in approximately 15%-30% of patients treated with intravenous artesunate, particularly those with high parasitaemia [13]. Monitoring haemoglobin, bilirubin, reticulocyte count, and LDH after discharge is therefore recommended in severe malaria cases.

The coexistence of parvovirus B19 and EBV likely significantly amplified the patient’s haemolytic process. Parvovirus B19 classically targets erythroid precursor cells within the bone marrow via the P antigen receptor, suppressing erythropoiesis. In patients with chronic haemolytic disorders, this may precipitate an aplastic crisis; however, parvovirus has also been associated with autoimmune haemolytic anaemia through molecular mimicry and complement activation [6]. In this patient, the elevated reticulocyte count suggested preserved marrow compensation rather than classic aplasia, supporting an immune-mediated mechanism. EBV is similarly associated with autoimmune haemolytic anaemia, most commonly cold agglutinin disease mediated by complement-fixing IgM antibodies [7]. The patient’s direct antiglobulin test positivity for C3d, with negative IgG, strongly supports complement-mediated haemolysis, characteristic of cold agglutinin disease. The detection of cold agglutinins further strengthens this interpretation.

Importantly, acute severe infections such as malaria may trigger reactivation of latent herpesviruses, including EBV, through transient immune dysregulation. Therefore, EBV seropositivity in this setting may represent either acute infection or viral reactivation. Distinguishing between the two can be difficult and often requires viral capsid antigen (VCA), EBV nuclear antigen (EBNA), and PCR correlation. RSV infection in adults is usually mild but can contribute to systemic inflammatory activation and cytokine release [8]. Although unlikely to independently explain the severity of illness, RSV may have compounded the inflammatory milieu already generated by malaria and the other viral infections.

The positive Lyme IgM with a negative IgG result was identified on immunoblot testing and was considered supportive of early Lyme disease in the appropriate clinical context. Isolated IgM positivity may occur during the early phase of infection prior to IgG seroconversion, particularly within the first few weeks of illness. Although false-positive Lyme IgM results are recognised during acute viral illnesses such as EBV and parvovirus infection due to polyclonal B-cell activation and immune dysregulation, the immunoblot findings in this case are diagnostic of true *Borrelia *exposure rather than simple cross-reactivity. It is possible that the patient acquired the *Borrelia *infection before travel or during transit through endemic regions. While Lyme disease alone was unlikely to explain the severity of presentation, it may have contributed to the overall inflammatory and immune-mediated response. This case highlights the importance of interpreting Lyme serology within the wider clinical and epidemiological context, particularly in patients with complex multisystem infectious presentations.

This case also offers practical lessons regarding the workup of persistent fever in travellers with confirmed malaria. Additional investigation should be considered when patients demonstrate persistent fever despite parasite clearance, disproportionate anaemia or haemolysis, persistent inflammatory marker elevation, new organ dysfunction, unexpected laboratory abnormalities, or respiratory and neurological symptoms unexplained by malaria. A structured diagnostic approach may include repeat malaria smears and parasite quantification; blood cultures and bacterial evaluation, viral PCR and serology (including EBV, CMV, parvovirus, HIV, and dengue); haemolysis workup including direct antiglobulin testing, haptoglobin, reticulocyte count, and cold agglutinins; tick-borne infection screening when epidemiologically relevant; and evaluation for hemophagocytic lymphohistiocytosis in patients with extreme hyperferritinaemia or cytopenias. In this case, the presence of complement-mediated haemolysis significantly altered management and prompted corticosteroid therapy alongside close haematology involvement.

Management of polymicrobial infection requires coordination across multiple specialities. Infectious disease input guided antimicrobial escalation and interpretation of complex serology, while haematology involvement was essential for evaluating immune-mediated haemolysis and transfusion support. Critical care and renal support may also become necessary in severe falciparum malaria complicated by organ dysfunction. The decision to initiate corticosteroids likely reflected concern for ongoing immune-mediated haemolysis rather than malaria itself. While corticosteroids are not routinely recommended in malaria, they may be appropriate when autoimmune haemolytic mechanisms coexist. This case ultimately demonstrates how overlapping infectious and immunological processes can interact synergistically, producing clinical severity greater than would be expected from any single pathogen alone.

## Conclusions

This case underscores the importance of considering coinfections in returning travellers, particularly when fever persists despite appropriate initial treatment. While malaria was the primary diagnosis, it did not fully explain the patient’s ongoing symptoms or the severity of her clinical course. The combination of malaria, concurrent viral infections, and possible Lyme disease created a highly complex presentation, with severe haemolysis as a dominant feature. The interaction between these conditions likely amplified the immune response, contributing to both diagnostic uncertainty and clinical severity.

This case highlights the need to maintain a broad and flexible diagnostic approach, especially in patients who fail to improve as expected. It also emphasises the importance of recognising overlapping disease mechanisms and the value of multidisciplinary care in managing complex infectious presentations.
